# Prenatal exposure to a mixture of non-persistent endocrine-disrupting chemicals and attention and hyperactivity symptoms in children in the ECHO cohort

**DOI:** 10.1016/j.envint.2026.110488

**Published:** 2026-08-29

**Authors:** Teresa Herrera, Sara Hyman, Roger Newman, Aisha S. Dickerson, Lesliam Quirós-Alcalá, Deborah H. Bennett, Jiwon Oh, Gredia Huerta-Montañez, Andrea R. Titus, Iván Díaz, Deborah J. Watkins, Leonardo Trasande, Brennan H. Baker, Assiamira Ferrara, Andrea E. Cassidy-Bushrow, Marnie Hazlehurst, Akhgar Ghassabian, for the Environmental influences on Child Health Outcomes (ECHO) Cohort Consortium

**Affiliations:** a NYU Grossman School of Medicine, Department of Population Health, New York, NY, USA; b NYU Grossman School of Medicine, Department of Pediatrics, New York, NY, USA; c Medical University of South Carolina, Charleston, SC, USA; d Johns Hopkins University, Bloomberg School of Public Health, Department of Epidemiology, Baltimore, MD, USA; e Johns Hopkins University, Bloomberg School of Public Health, Department of Environmental Health and Engineering, Baltimore, MD, USA; f UC Davis School of Medicine, Department of Public Health Sciences, Division of Environmental and Occupational Health, Davis, CA, USA; g Northeastern University, Department of Civil and Environmental Engineering, Boston, MA, USA; h University of Michigan School of Public Health, Department of Environmental Health Sciences, Ann Arbor, MI, USA; i University of Washington & Seattle Children’s Research Institute, Seattle, WA, USA; j Kaiser Permanente, Division of Research, Oakland, CA, USA; k Henry Ford Health System, Public Health Sciences, Detroit, MI, USA; l Seattle Children’s Research Institute, Seattle, WA, USA

**Keywords:** Prenatal exposure, Chemical mixtures, Neurodevelopment, Child behavioral checklist

## Abstract

**Objective:**

To examine the association between prenatal exposure to a mixture of EDCs and attention-deficit/hyperactivity disorder (ADHD) symptoms in children.

**Methods:**

Participating were mother–child pairs from the Environmental influences on Child Health Outcomes (ECHO) Cohort (n = 3,962, 18 Sites, 2001–2021). Maternal spot urine samples collected during pregnancy were sent to the Wadsworth Center Human Health Exposure Analysis Resource laboratory and analyzed using a single assay workflow. From those samples, 24 urinary metabolites of EDCs with detection frequency > 70%, were grouped as molar sum of high- and low-molecular-weight phthalates, polycyclic aromatic hydrocarbons, bisphenol S, organophosphate esters, and organophosphate and pyrethroid pesticides. Child ADHD symptoms were assessed using raw scores from the Child Behavior Checklist for preschool-aged (CBCL/1½-5) and school-aged (CBCL/6–18) Attention-Deficit/Hyperactivity Problems subscale. Scores ≥ the 85th and 95th percentiles were respectively classified as borderline and clinical thresholds. Quantile g-computation estimated the expected change in ADHD outcome associated with a one-quartile increase in all prenatal EDC mixture concentrations, using negative binomial models for raw scores and binomial models for percentile thresholds.

**Results:**

Prenatal exposure to the EDC mixture was not associated with child ADHD symptoms in preschool-aged or school-aged children in adjusted models for either continuous raw scores or percentile thresholds.

**Conclusion:**

Overall our findings on prenatal EDC mixture exposure and ADHD symptoms were not robust to full adjustment. Future research that can delineate factors such as windows of vulnerability and trajectories of ADHD-related symptoms may help clarify mixed findings in the literature.

## 1. Introduction

Attention-Deficit/Hyperactivity Disorder (ADHD) is a neurodevelopmental condition defined by persistent inattention, hyperactivity, and impulsivity symptoms that can impact daily functioning, health, educational experiences, and social relationships, particularly in the absence of treatment and adequate accommodation (National Institute of Mental Health, 2024). In 2022, the estimated number of children aged 3–17 years old diagnosed with ADHD in the US was 7.1 million (Danielson et al., 2024). ADHD’s etiology is complex, involving genetic, epigenetic, environmental, and neurobiological factors, and can vary across individuals (Poddar et al., 2025). A heritability estimate of 60–90% highlights an opportunity to intervene at the population level by reducing exposure to environmental factors that contribute to the etiology (Thapar, 2018). Addressing environmental risk factors is particularly important as ADHD symptom presentation may result from exposure to understudied neurotoxicants.

Endocrine-disrupting chemicals (EDCs) like polycyclic aromatic hydrocarbons (PAHs), organophosphate pesticides and pyrethroids, organophosphate ester flame retardants (OPEs), phthalates, and bisphenols, have emerged as potential environmental risk factors for ADHD (Kim et al., 2022; Bennett et al., 2016). Broadly, EDCs disrupt hormone function and contribute to disease across multiple organ systems, including the central nervous system (Ghassabian et al., 2022). While these chemicals have short half-lives in the human body, their influences on the endocrine system and other organs can be long-lasting (Gore et al., 2015). Pregnancy is a particularly vulnerable period for exposure since EDCs can cross the placental barrier and impact organs, such as the brain (Ghassabian et al., 2022). Prenatal exposure may disrupt neurodevelopmental processes by disrupting thyroid function, altering neuronal connectivity, generating oxidative stress, and influencing epigenetic modifications (Dickerson et al., 2023). Given the widespread exposure to EDCs in the environment, there is an urgent need to better understand the potential neurotoxic effects of EDCs during sensitive developmental periods such as pregnancy.

Multi-chemical approaches to EDC research are especially important given that single-chemical risk assessment can underestimate chemical risks (Kortenkamp and Faust, 2018). Individuals are commonly exposed to mixtures of chemicals, so such mixtures should be incorporated into research on their health effects. Currently, studies on EDC mixtures and ADHD symptoms are sparse (Oh et al., 2023). While a prior study detected associations between composite PAH exposure during pregnancy and ADHD-related outcomes, few have incorporated PAHs into broader prenatal mixture analyses that reflect real-world co-exposure to multiple chemical classes with potentially similar downstream effects (Perera, et al., 2014). Longitudinal studies have also shown that exposure to certain phthalates are associated with ADHD-related outcomes, depending on the molecular weight of the compound and child sex (Minatoya and Kishi, 2021). Emerging classes of contaminants, such as OPEs, which are increasingly used as flame-retardants and plasticizers, have seldom been included in multichemical mixture studies despite evidence from a 2025 study in the Environment and Child Health Outcome (ECHO) cohort showing associations of prenatal exposure to some OPEs with externalizing behavior in preschool-aged children (Oh et al., 2025). Few studies have evaluated exposure to mixtures of various classes of EDCs and ADHD outcomes in children (Oh et al., 2023).

We aimed to examine associations between exposure to an EDC mixture during pregnancy and offspring ADHD symptoms. We used data from mother-child pairs participating in the ECHO Cohort in the US (Knapp et al., 2023). Rather than focusing on individual chemical families, our overarching goal was to evaluate the combined impact of concurrent exposure to highly detected non-persistent EDCs during pregnancy on child neurodevelopment. We included a wide range of chemical classes previously reported to influence offspring neurodevelopment, reflecting real-world co-exposure. In addition, we evaluated several exploratory analyses stratified by variables like age, sex, and neighborhood conditions, as certain communities experience a higher cumulative burden to multiple EDC classes (Nguyen et al., 2020). We hypothesize that higher exposure to a mixture of EDCs during pregnancy will be associated with more ADHD symptoms.

## 2. Methods

### 2.1. Study population

The study population included mother–child pairs participating in the ECHO Cohort who had at least one maternal urine sample collected during pregnancy to measure chemical concentrations, and symptom measures based on either the preschool or school versions of the Child Behavior Checklist (CBCL) (n = 4,044). The ECHO cohort is a large diverse prospective pediatric cohort study with ongoing recruitment across 50 U.S. states, the district of Columbia, and Puerto Rico (Knapp et al., 2023). ECHO aims to advance understanding of how early life exposures influence child health and uses a unified study protocol to collect detailed information on environmental exposures and various outcomes (Knapp et al., 2023). A full description of the cohort design and study protocol have been published previously (Knapp et al., 2023; Blackwell et al., 2024). For families with siblings, one sibling was randomly included, to avoid non-independence of observations. Our final analytical sample included 3,962 mother–child pairs from 18 ECHO cohort sites between 2001–2021 (Supplementary Fig. 1).

### 2.2. Exposure

Urinary samples provided during pregnancy at ECHO cohort sites were sent to the Wadsworth Center Human Health Exposure Analysis Resource (HHEAR) laboratory. Samples were analyzed for analytes from ten chemical groups using solid phase extraction coupled with liquid chromatography-tandem mass spectrometry (Buckley et al., 2022). Urine specimens were analyzed using a single assay workflow at one HHEAR laboratory, as previously described by (Buckley et al., 2026). Samples were randomized to minimize cohort-site and batch effects (Buckley et al., 2026). Analytical accuracy was assessed using standard reference materials and quality control spikes, and reproducibility was assessed using quality control pools and duplicate samples (Buckley et al., 2026). Detailed methods and full quality control results are provided in the supplementary materials accompanying (Buckley et al., 2026). The analytical method has been published previously by (Zhu et al., 2021).

Due to their potential for neurotoxicity, we focused on phthalates, bisphenols, OPEs, a total pesticides group (organophosphate pesticides and pyrethroids), and PAHs in this analysis (24 analytes, Supplementary Table 1). Most participants (98%) had one chemical measurement during pregnancy. When a participant had multiple measures during pregnancy (n = 87 or ~ 2%), we prioritized those from the second trimester, which was the most common in our analytic sample.

To create the mixture, we first checked the proportion of values below the limit of detection (LOD) for each analyte. If the proportion of concentrations below LOD or non-detects was 30% or less, missing values were replaced by the LOD/sqrt2 for each specific analyte (Hornung and Reed, 1990). Of the 68 analytes originally considered for analysis, 44 were excluded because more than 30% of values were below the LOD. A full list of study analytes, their geometric means and LOD detection frequencies are presented in Supplementary Table 1. A 30% non-detect threshold was chosen to ensure the majority of the sample consisted of measured data and avoid over imputation. After imputation of values below the LOD, analyte concentrations were converted to molar units using the analyte’s molecular weight. These concentrations were then summed into molar sum groups based on shared characteristics, including high molecular weight (HMW) phthalates, low molecular weight (LMW) phthalates, bisphenol S (BPS), OPEs, pesticides, and PAHs as 6 distinct mixture components. Di-ethyl-hexyl phthalate (DEHP) metabolites were summed prior to being incorporated into the HMW phthalate group (Supplementary Table 1). Each summed group was considered as a component of the mixture. We accounted for urinary dilution using specific gravity (sg) with the following formula (Kuiper et al., 2022; Boeniger et al., 1993).


$$adjustedconcentration=molarsumconcentration\times \frac{\left(medianbatchsg\;-\;1\right)}{\left(individualsg\;-\;1\right)}$$

Following dilution adjustment, molar sum groups were natural log-transformed to normalize skewed distributions.

### 2.3. Outcome

Child ADHD symptoms were assessed using the Diagnostic and Statistical Manual of Mental Disorders (DSM)-oriented Attention Deficit/Hyperactivity Problems subscale from the CBCL/1½-5 and CBCL/6–18. The CBCL is a component of the Achenbach System of Empirically Based Assessment (ASEBA) that uses parent-report of children’s emotional and behavioral problems in preschoolers (CBCL/1½-5) and school-aged children (CBCL/6–18) (Achenbach and Rescorla, 2000; Achenbach and Rescorla, 2001). It has been validated and the ADHD subscale has high diagnostic accuracy for identifying potential ADHD across diverse populations (Aebi et al., 2010; Stewart et al., 2024). If a child had multiple CBCL records, we opted for the school age version or the most recent record. In our statistical analysis, we opted for the use of the raw scores, as T-scores are truncated at the lower end of the distribution, thereby reducing variability in the outcome (Thurber and Sheehan, 2012). We used distribution-based thresholds to categorize CBCL raw scores into elevated symptom groupings. Scores that were ≥ the 85th percentile were classified as borderline clinical symptoms and scores ≥ the 95th percentile were classified as clinically elevated symptoms. The CBCL/1½–5 and CBCL/6–18 DSM-oriented ADHD Problems subscales differ in item content and number. Therefore, results are presented stratified by form. Finally, we explored dichotomized CBCL symptom T-scores as < 65 (non-elevated) vs. ≥ 65 (borderline clinical), and physician’s ADHD diagnosis as reported by the parent or caregiver as secondary outcomes. While physician ADHD diagnosis has the greatest clinical relevance, 61.5% of participants lacked these data; therefore, we considered it a secondary outcome.

### 2.4. Covariates

Covariates were selected based on the literature and a Directed Acyclic Graph of the study question (Supplementary Fig. 2). Sociodemographic, anthropometric, and medical history characteristics were gathered from ECHO data collection forms and/or extracted from medical records, harmonized across sites by the ECHO Data Analysis Center (Knapp et al., 2023). The covariates selected were ECHO cohort site, maternal age at delivery (continuous), child’s family history of a first degree relative diagnosed with a psychiatric condition (e.g. ADHD, autism, depression), maternal race (American Indian, Asian, Black, Multiracial, Pacific Islander, or White), maternal ethnicity (Hispanic vs. Non-Hispanic), maternal education (high school – no diploma, high school diploma or General Education Development or GED program, some college education or an associate’s degree, or a bachelor’s degree or more), annual household income (<$50,000 vs. ≥$50,000), body mass index (BMI) category (underweight, average weight, overweight, obese), maternal smoking (smoked vs. did not smoke) and alcohol use (drank vs. did not drink) during pregnancy, and parity (nulliparous vs. parous).

### 2.5. Statistical analysis

In descriptive analyses, we characterized categorical and continuous variables by calculating counts and percentages, and means and standard deviations (SD), respectively. Additionally, we calculated summary statistics of the molar sum groups in the chemical mixture. We measured correlation patterns using Spearman correlation coefficients and depicted the results in a heat map. To aid in model specification, we plotted distributions and checked for linear model assumptions by examining residuals.

Analyses were performed using quantile g-computation to estimate the mixture effects under the following counterfactual scenario in which all participants were exposed to a one-quartile increase in all exposures simultaneously (Keil et al., 2020). We selected quantile g-computation given its ability to accommodate over dispersed outcomes and mixture components with effects in different directions, as well as its lower computational burden relative to other methods (Keil et al., 2020). In the primary analysis, we assessed a mixture of all six molar-sum groups, rather than assessing mixtures of each chemical class separately. Given that the CBCL/1½-5 and CBCL/6–18 raw scores were skewed, discrete, and bounded, we tested for zero inflation and afterward decided to use a negative binomial regression within a hurdle framework as implemented in the *qgcomp* R package (Keil, 2025). The hurdle model separated the outcome into two components. First, the probability of having a zero score (no symptoms) and second, the expected change in raw score among children with non-zero scores. For raw score percentile thresholds, T-score threshold, and ADHD diagnosis, we used binomial regression within the quantile g-computation framework.

Confounders and precision variables included maternal age during pregnancy, family history of any psychiatric disorder, race, ethnicity, parity, BMI category, education, income, child sex, child age at assessment, and cohort site. For models assessing T-scores, we omitted child sex and age from the model equations.

To examine the contribution of individual mixture components, we first considered weights provided by quantile g-computation, which are interpreted as the proportion of the negative or positive partial effect contributed by each mixture component (Keil et al., 2020). These weights can be used to understand how individual components contribute to the mixture’s overall effect. However, when the mixture effect is null, the weights are less interpretable. Therefore, we presented both the mixture weights and ran separate multivariate models for each mixture component using negative binomial and binomial regression to examine the relationship between CBCL/1½-5 and CBCL/6–18 raw scores and each molar sum group. Because we used multiple imputation, the weights presented represent the median across the distribution of weights from models run on imputed datasets. Weights and models were used to help interpret the contributions of individual components in the mixture.

Since our main analysis used molar-sum groups as mixture components, we also fitted a decomposed mixture model. In this model, the individual analytes that comprised the molar sum groups served as separate mixture components within a single joint mixture model to assess whether aggregation into molar sum groups affected the findings. We also present model weights for the un-summed analyte mixture as a butterfly plot.

We ran several exploratory stratified analyses. First, to explore age sensitivity, we conducted an age-stratified analysis. For CBCL/1½–5, age was dichotomized into 1½–3 year olds (n = 840) and 4–5 year olds (n = 1102). For CBCL/6–18, age was dichotomized into 6–9 year olds (n = 1599) and 10–18 year olds (n = 421).We also examined 2nd and 3rd trimester exposures separately to assess the potential for trimester-specific effects. However, 1st trimester sample sizes were too small for separate CBCL analyses, and formal vulnerability-window testing was not possible without longitudinal exposure measures. We also considered sex-stratified analyses. To explore the potential role of neighborhood conditions in the relationship, stratified models were run for child opportunity index (COI) at residential addresses at birth. The COI is a composite index of neighborhood characteristics that support healthy child development (Acevedo-Garcia et al., 2020). The COI includes measures that capture social, economic, health, environmental, and education conditions within communities (Acevedo-Garcia et al., 2020). We used 2015 COI 2.0 scores categorized into standardized percentile rankings, into the five levels of opportunity: very low (<20th percentile), low (20th to < 40th), moderate (40th to < 60th), high (60th to < 80th), and very high (≥80th percentile) (Aris et al., 2022).

To ensure robustness of models, several sensitivity analyses were conducted. Because many sociodemographic variables like education, income, race, and ethnicity can function as either confounders or effect modifiers, and behaviors like smoking and alcohol use had low reported prevalence, we ran a minimally adjusted model set excluding these covariates to better isolate their role. We also conducted a complete-case analysis and an analysis excluding cohort sites with fewer than 100 participants.

All analyses were performed using R 4.5.0. and missingness of covariates was addressed using multiple imputation using chained equations. Mixture models were fit separately on 20 imputed datasets and results were pooled using Rubin’s rules (Rubin DB, 1987).

## 3. Results

### 3.1. Descriptive results

Our study population, described in Table 1, included 3,962 mother–child pairs from 18 cohort sites, with contributions ranging from 25 to 682 mother–child pairs. The average age of mothers was 30.1 years (SD = 5.6) at the time of pregnancy. Most mothers included in the study were non-Hispanic (75.5%) and White (53.6%), and had at least some college education (63%). Most participants reported not smoking (79.8%) or drinking (64%) during pregnancy. Approximately half of children had CBCL/1½-5 (preschool) and the rest CBCL/6–18 (school aged) version. The average age of children at the time of the CBCL assessment was 6.26 years old and 50.5% of the children in the study were male. Across both CBCL/1½-5 and CBCL/6–18 surveys, 20.1% had ADHD raw scores ≥ 85th percentile, and 6% had scores ≥ 95th percentile. Proportions ≥ the 85th and 95th percentiles exceeded 15% and 5%, respectively, due to tied values at the cutoff. Thirty-four percent of the children had a first-degree relative who had been diagnosed with depression, anxiety, autism, or ADHD. A table of participant characteristics across the two forms can be found in Supplementary Table 2.

Most urine samples were provided during the second trimester (n = 2,078), followed by the third (n = 1510) and first (n = 374). The correlation between molar sum groups ranged from -0.03 to 0.43 (Supplementary Fig. 3), with LMW and HMW phthalates being most strongly correlated. Additional chemical group characteristics are reported in Supplementary Table 3.

### 3.2. Associations between the EDC mixture and CBCL raw scores

The unadjusted and adjusted joint effects of the chemical mixture on ADHD symptoms from hurdle models are presented in Fig. 1. In unadjusted models, a quartile increase in mixture exposure was associated with a increased CBCL raw scores in preschool aged children (OR = 1.11, 95%CI 1.04, 1.17). This changed in fully adjusted models, where in the count portion of the model, we did not find any associations between the mixture and CBCL raw scores. Null results were observed across preschool-aged (RR = 1.02, 95% CI 0.96, 1.09) and school-aged (RR = 0.96, 95%CI 0.88, 1.05) children. In the hurdle component of fully adjusted model, we did not find any association between exposure to a mixture of EDCs and odds of having a raw score of zero. Findings were similar with preschool-aged (OR = 0.92, 95%CI 0.75, 1.14) and school-aged (OR = 0.98 95%CI 0.80, 1.19) children. Additionally, when examining the association between individual analytes as mixture components, rather than molar-sum groups, and raw scores, the results were consistent with our main analysis (Supplementary Table 4).

### 3.3. Associations between the EDC mixture and CBCL raw score thresholds

When we looked at associations between the mixture and CBCL raw scores above percentile cutoffs, results were largely null (Fig. 2). In unadjusted models, there were associations between exposure to the mixture and having a score ≥ 85th percentile (OR: 1.30, 95%CI: 1.08–1.58) and ≥ the 95th percentile (OR: 1.51, 95%CI: 1.05–2.18) in preschool-aged children but not school aged. In fully adjusted models results were null for both preschool and school aged children. We did not observe significant associations in analyses exploring a T-score borderline/clinical cutoff (Supplementary Table 5). Finally, for those with data on ADHD diagnosis, no associations were found between the mixture and parent-reported physician-diagnosed ADHD (OR: 1.06, 95% CI: 0.78, 1.45) (Supplementary Table 5).

### 3.4. Mixture component contributions

Table 2 presents single-molar sum group multivariate models, evaluating associations between separate molar sum groups and ADHD symptom raw scores. Each chemical group was examined independently in both preschool- and school-aged children. While, there were positive associations between HMW phthalates and preschool-aged (RR = 1.02, 95%CI = 0.97, 1.08) and school-aged (RR = 1.03, 95%CI = 0.97, 1.09) raw scores, both models were statistically insignificant (Table 2). We also did not observe significant associations between individual molar sum groups with raw scores. Other chemical groups were not associated with raw scores or percentile thresholds in single-exposure examination of chemical molar sum groups. (Supplementary Table 6).

HMW phthalates showed the largest positive weights in count portions of our main models (Supplementary Figs. 4 and 5). In the decomposed analyte mixture models, different HMW phthalate metabolites like Mono-2-(propyl-6-carboxy-hexyl)-phthalate (MPCHP) and Mono (3-carboxypropyl) phthalate (MCPP) had the largest positive weight at preschool-age and Monohydroxy-*iso*-nonyl phthalate (MHINP) and Mono-benzyl phthalate (MBZP) at school-age. (Supplementary Figs. 6 and 7). However, given the null overall mixture findings, these weights should be interpreted with caution.

### 3.5. Secondary and sensitivity analyses

Adjusted stratified models presented in supplementary material were largely null. In age stratified analyses, results were not statistically significant in any age group across both model components (Supplementary Table 7). Trimester-specific analyses, associations remained null across both trimester 2 and trimester 3 for preschool and school-aged CBCL outcomes (Supplementary Table 8). For example, in the count component, associations were null in trimester 2 for preschool (RR = 1.06, 95%CI; 0.96, 1.16) and school-aged (RR = 0.94, 95%CI; 0.84, 1.03) forms, and in trimester 3 for preschool (RR = 0.92, 95%CI; 0.82, 1.03) and school-aged (RR = 1.06, 95%CI; 0.91, 1.23) forms. (Supplementary Table 8). Null associations were also observed in stratified models by sex and COI (Supplementary Tables 9 and 10).

In minimally adjusted models (Supplementary Fig. 8), the association in the count portion of the model for preschool aged children strengthened relative to full adjustment (minimum adjustment RR = 1.06, 95%CI; 1.00, 1.14 versus fully adjusted RR = 1.02, 95%CI; 0.96, 1.09). Running a sensitivity analysis leaving out smaller sites, did not materially affect our results (Supplementary Table 11). Complete case analysis results were directionally consistent to main analysis. However, effect confidence intervals were wider across estimates, likely due to reduced sample size. (Supplementary Table 12).

## 4. Discussion

### 4.1. Study overview

In this large prospective cohort study in the US, we did not observe a joint effect of prenatal exposure to EDC mixture, composed of phthalates, bisphenols, OPEs, organophosphate pesticides and pyrethroids, and PAHs, on ADHD symptoms. Results were also null in the subsample of participants with parent-reported ADHD diagnosis. Examination of chemicals in individual molar sum group models showed positive but statistically insignificant associations between prenatal HMW phthalate exposure and CBCL ADHD symptom raw scores.

### 4.2. Comparison to prior literature

Our study contributes to the limited literature exploring prenatal chemical mixtures and childhood ADHD symptoms. A 2021 nested case-cohort study by Choi et al. reported a reduction in prenatal OPE- and phthalate mixture was associated with decreased ADHD risk (Choi et al., 2021). Choi and colleagues used ADHD diagnoses from the Norwegian Patient Registry. Differences in study design, timing of recruitment, population homogeneity, and healthcare systems in Norway may have contributed to discrepant findings. Compared to Choi et al., we evaluated a broader mixture of highly detected analytes represented as molar sum groups and used a quantitative measure of ADHD symptoms in addition to the parent-report of a physician diagnosis of ADHD. A 2025 study (n = 1,658) by Fabbri et al. evaluated mixtures of per- and polyfluoroalkyl substances, metals, phenols, phthalates, and organophosphate pesticides, none of which were associated with behavioral problems (Fabbri et al., 2025). In single analyte models, prenatal bisphenol A (BPA) was associated with CBCL/6–18 attention problem scores in adolescence (Fabbri et al., 2025). Our analysis included bisphenol S, a newer replacement of BPA, which was detected at a higher rate compared to BPA in the ECHO Cohort. A Dutch cohort study (n = 782) examining prenatal exposure to phthalates, bisphenols, and organophosphate pesticides, found a null joint mixture effect on attention-related behavioral outcomes using quantile g-computation (Van Den Dries et al., 2021). However, a French cohort study (n = 416) by Guilbert et al. reported an association between a phenol and phthalate mixture and CBCL/1½-5 externalizing scores in preschool aged girls, with monoethyl phthalate (MEP) being a key contributor (Guilbert et al., 2021). Meanwhile, a 2025 ECHO cohort study, observed associations between a phenol and phthalate mixture and CBCL/1½-5 externalizing scores in preschool aged children (n = 2,617), with MEP and a HMW phthalate metabolite being contributors (Nakiwala et al., 2025). In our study, LMW phthalates were not associated with our outcome. These discrepancies may reflect differences in phenotypes represented by CBCL externalizing scales vs. ADHD symptom scales, population differences in exposure levels, or differences in the components of mixture EDCs. Compared to the studies mentioned above our study had the largest sample size and widest age range, examining both ADHD symptoms and ADHD diagnosis (even though in a subsample), and including a wide range of chemical compounds with potentially similar downstream effects in the mixture. The associations observed with externalizing symptoms in other studies but not ADHD symptoms in our study may reflect that prenatal EDC exposure does not necessarily lead to an ADHD-specific phenotype but something slightly different.

PAHs are sparsely included in mixture analysis and ADHD symptoms, even though the underlying mechanisms of effects could be very similar to other EDCs, such as thyroid disruption (Park et al., 2024). Focused only on PAHs, a cohort study in NYC found prenatal PAH–DNA adducts were not associated with CBCL symptom scores, though they were linked to ADHD-related subscales from the Conners Parent Rating Scale (Perera, et al., 2014). Another study from the same cohort reported more ADHD symptoms among children with both high prenatal summed PAH exposure and persistent material hardship (Perera et al., 2018). This work also underscores the added complexities of considering socioeconomic context in neurodevelopmental research. While we explored COI, an in-depth analysis of the role of social disadvantage was beyond the scope of this paper. Nonetheless, future studies should aim to shed light on the social dimensions of mixture research.

Several analytes and analyte groups examined in our mixture models have been associated with ADHD-related outcomes in the single-chemical analyte and group literature. For instance, the summed PAH group from Perera et al. associated with ADHD symptoms included several HMW PAHs, that were not detected in our sample and recruitment was between 1998–2006 (Perera et al., 2018). An analysis from the HOME Study in Ohio (n = 223) found gestational OPE exposure, particularly bis(2-chloroethyl) phosphate (BCEP) at 26 weeks, associated with poorer executive function scores in adolescence. BCEP was not measured in our study (Vuong et al., 2024). BPS, included as the single bisphenol component in our mixture, was associated with higher childhood ADHD symptoms in Kim et al. particularly among girls at age 6, whereas we did not observe an association with BPS in our study (Kim et al., 2022). We looked at a much wider age range, than Kim et al. Differences in detected analytes between studies may have contributed to our overall null effect which could have been driven by more contemporary exposure represented in our study.

### 4.3. Study strengths and limitations

Our study has several strengths, as we were able to examine a unique mixture to capture the joint burden of highly detected analytes during pregnancy. Our study benefits from its large sample size and unified protocols across varying cohort sites. In addition, all analytes were measured and assayed in the same laboratory, reducing measurement error. We were also able to examine multiple outcomes capturing symptom severity, thresholds, and parent-reported diagnosis, and explored these outcomes in both preschool and school aged children. While our study did not evaluate a specific biologic or policy-defined shift in exposure, it provides an estimate of relatively higher versus lower exposure to multiple EDCs, better reflecting real world exposure to highly detected analytes in our population.

Our study contained several limitations. First, exposure was assessed at different gestational time points, which may limit interpretation if gestational timing reflects biologically distinct exposure states and periods of susceptibility to different chemicals. While urine is commonly used for non-persistent analyte detection, single spot urine samples and the short half-lives of these compounds may not be fully representative of exposure over the course of pregnancy. In addition, the CBCL is a screening tool that captures behaviors consistent with ADHD-like symptoms but is not diagnostic. The CBCL/1½-5 ADHD subscale is less reliable in the preschool age group, and symptom scores should be interpreted with caution. However, screening tools like CBCL can capture populations that are less likely to access diagnostic testing. We also were unable to determine whether children in our study had received behavioral or medical interventions, or whether parental knowledge of ADHD may have influenced reporting. Also, prenatal EDC exposures may have age-dependent effects. While formal evaluation of symptom trajectories was beyond the scope of this study, it is an important direction for future research. We did not observe sex-specific effects, which may reflect how the mixture operates similarly across sexes. However, it is possible that disruption of certain sex steroids is relevant to specific exposure windows, and jointly stratifying by timing and sex was not feasible given power constraints.

### 4.4. Conclusion

In sum, this large study in a nationwide sample from the US showed no joint effect of EDC mixture on ADHD symptoms in children. Our study underscores the need for future research on EDC mixtures to better tease apart the roles of gestational timing, the combined effects of social stressors, and broader exposure profiles.

## Supplementary Material

1

2

## Figures and Tables

**Fig. 1.**
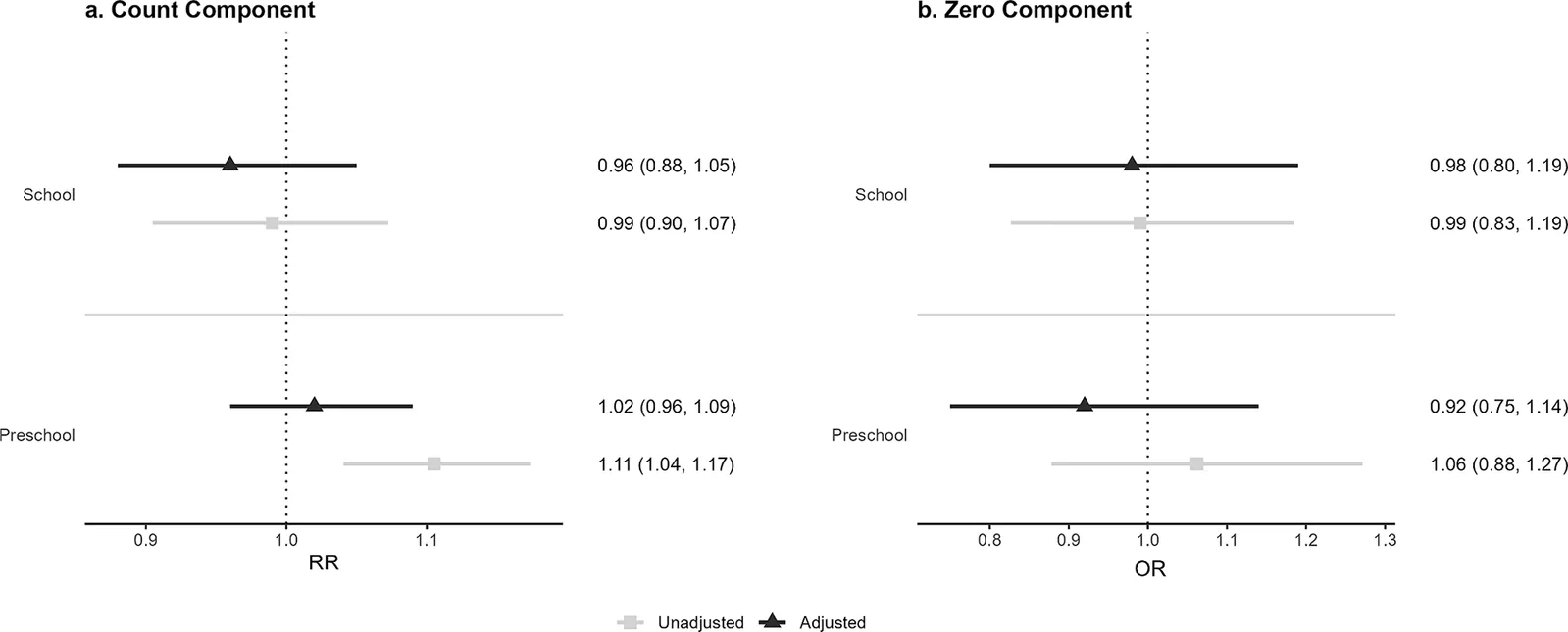
Associations between prenatal exposure to a mixture of endocrine-disrupting chemicals and Attention Deficit and Hyperactivity Problem Scores. RR = rate ratio for ADHD raw score among children with a non-zero score per one-quartile joint increase in all exposures in the mixture derived from negative binomial hurdle mixture models. OR = odds ratio for the likelihood of exhibiting ADHD symptoms (score not = 0) per one-quartile joint increase in all exposures in the mixture derived from negative binomial hurdle mixture models. All models adjusted for maternal age, body mass index, education, parity, pregnancy smoking and alcohol use, race, ethnicity, and child sex and age at CBCL assessment, family history of psychiatric illness, and cohort site. Analyses were conducted separately among preschool-age (n = 1,942) and school-age (n = 2,020) children. Mixture components included 6 summed groups of: OPEs, organophosphate esters; Pesticides organophosphate pesticides and pyrethroids; PAH, polycyclic aromatic hydrocarbons; HMW Phthalates, high-molecular-weight; LMW Phthalates, and Bisphenol S. Chemicals were adjusted for specific gravity and log transformed. ADHD, attention deficit and hyperactivity disorder; CBCL, Child Behavior Checklist; CI, confidence interval; RR, rate ratio; OR, odds ratio.

**Fig. 2.**
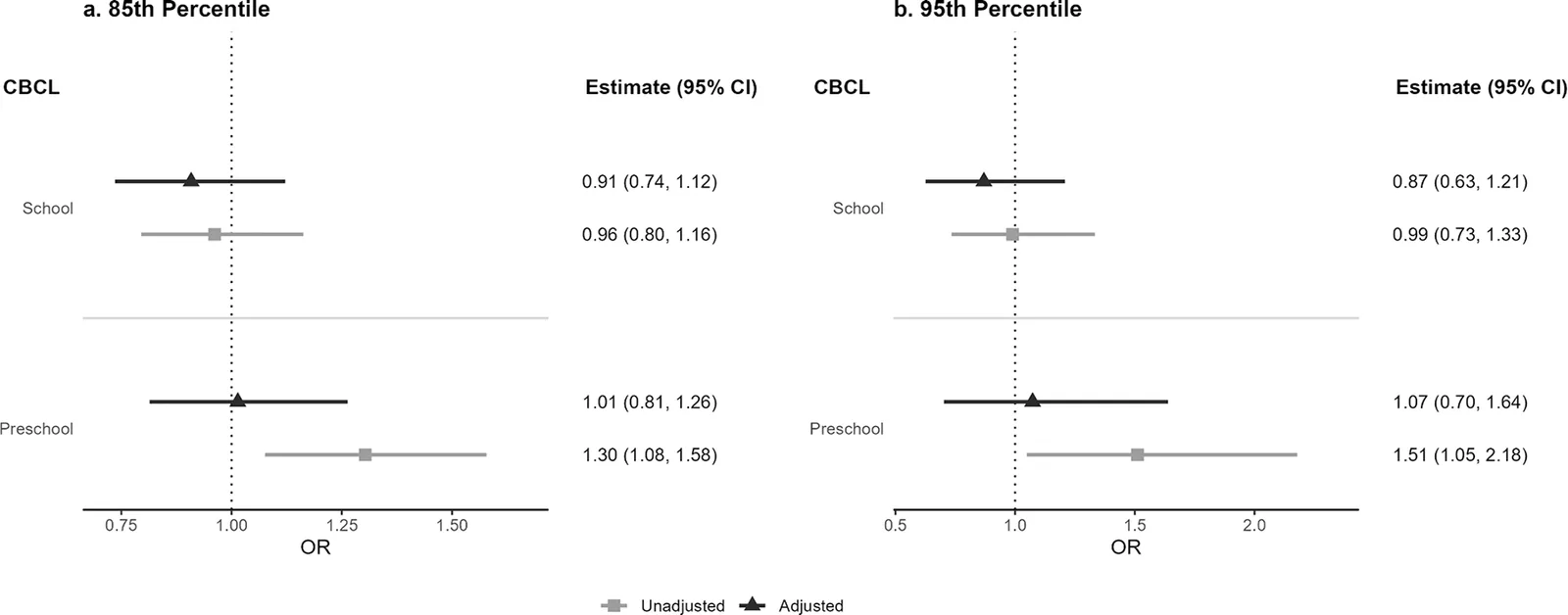
Associations between prenatal exposure to a mixture of endocrine-disrupting chemicals and CBCL Attention Deficit and Hyperactivity Problem Subscale Raw Scores Percentiles Thresholds. OR = odds ratio for the likelihood of scoring above the 85th and 95th percentiles, respectively, per one-quartile joint increase in all exposures in the mixture, derived from binomial logistic mixture models. All models were adjusted for maternal age, body mass index, education, parity, smoking and alcohol use during pregnancy, race, ethnicity, child sex and age at CBCL assessment, family history of psychiatric illness, and cohort site. Analyses were conducted separately among preschool-age (n = 1,942) and school-age (n = 2,020) children. Mixture components included 6 summed groups of: OPEs, organophosphate esters; Pesticides organophosphate pesticides and pyrethroids; PAH, polycyclic aromatic hydrocarbons; HMW Phthalates, high-molecular-weight; LMW Phthalates, and Bisphenol S. Chemicals were adjusted for specific gravity and log transformed. ADHD, attention deficit and hyperactivity disorder; CBCL, Child Behavior Checklist; CI, confidence interval; OR, odds ratio.

**Table 1:** Study sample characteristics (n = 3962).

*Maternal Characteristics*		

Maternal Age, year	Mean (SD)	30.1 (5.6)

Parity, n (%)	Parous	2255 (56.9)
Ethnicity, n (%)	Hispanic	836 (21.1)
Race, n (%)	American Indian	15 (0.4)
	Asian	201 (5.1)
	Black	815 (20.6)
	Multiple Race	166 (4.2)
	Pacific Islander	19 (0.5)
	White	2124 (53.6)
Education, n (%)	High school or less	1012 (25.5)
	Some college or above	2497 (63.0)
Income, n (%)	<$50,000	1395 (35.2)
	$50,000 +	1524 (38.5)
Smoking during pregnancy, n (%)	No	3165 (79.9)
Any alcohol use during pregnancy, n (%)	No	2535 (64.0)
Body mass index, n (%)	Underweight	104 (2.6)
	Average	1624 (41.0)
	Overweight	939 (23.7)
	Obese	1030 (26.0)
*Child characteristics*		
Child sex, female		1962 (49.5)
Age at CBCL survey, years	Mean (SD)	6.26 (3.06)
Family History Psychiatric illness	Yes	1384 (34.9)
*Outcome characteristics*		
CBCL Type, n (%)	Preschool	1942 (49.0)
	School	2020 (51.0)
CBCL Raw Score	Median [IQR]	2.00 [1.00, 5.00]
CBCL Raw Score P85, n (%)	≥ 85th percentile	797 (20.1)
CBCL Raw Score P95, n (%)	≥ 95th percentile	237 (6.0)
CBCL T-score Cutoff	≥ 65	501 (12.6)
Parent Reported ADHD Diagnosis	Yes	194 (4.9)

Notes: For selected dichotomous variables (parity, ethnicity, any alcohol use during pregnancy, smoking during pregnancy, family history of psychiatric illness, child sex, raw score percentiles, and ADHD diagnosis), only one category and missingness are presented for brevity. Family history of psychiatric illness includes any first-degree relative diagnosed with depression, anxiety, autism, or ADHD. CBCL indicates Child Behavior Checklist. ADHD diagnosis refers to a prior physician diagnosis of ADHD as reported by the caregiver.

Missing data (n (%)): education, n = 453 (11.4%); race, n = 622 (15.7%); ethnicity, n = 178 (4.4%); parity, n = 221 (5.6%); any alcohol use during pregnancy, n = 866 (21.9%); smoking during pregnancy, n = 557 (14.1%); income, n = 1043 (26.3%); Body mass index, n = 265 (6.7%); family history of psychiatric illness, n = 1443 (36.4%); parent-reported ADHD diagnosis, n = 2437 (61.5%).

**Table 2:** Individual Mixture Components and Child ADHD Symptoms.

	Preschool-aged	School-aged

Molar sum group	RR (95%CI)	
HMW Phthalates	1.02 (0.97, 1.08)	1.03 (0.97, 1.09)
LMW Phthalates	1.00 (0.96, 1.04)	0.98 (0.93, 1.03)
Bisphenol S	0.97 (0.92, 1.02)	0.99 (0.93, 1.04)
PAH	1.01 (0.96, 1.07)	0.98 (0.94, 1.02)
OPEs	1.01 (0.96, 1.05)	1 (0.94, 1.05)
Pesticides	0.99 (0.94, 1.03)	0.95 (0.89, 1.01)

RR = rate ratio from multivariate negative binomial regression representing the expected change in ADHD symptoms associated with a unit increase in each individual chemical group, accounting for cohort differences.

All 6 models were run separately and adjusted for maternal age, body mass index, education, parity, smoking and alcohol use during pregnancy, race, ethnicity, child sex and age at CBCL assessment, family history of psychiatric illness, and cohort site. Analyses were conducted separately among preschool-age (n = 1,942) and school-age (n = 2,020) children ADHD symptoms were derived from the ADHD subscales of the CBCL (preschool and school-age versions). Chemicals were adjusted for specific gravity and log transformed.

ADHD, attention deficit and hyperactivity disorder; CBCL, Child Behavior Checklist; CI, confidence interval; HMW, high molecular weight; LMW, low molecular weight; OPEs, organophosphate esters; PAH, polycyclic aromatic hydrocarbons; Pesticides, organophosphate pesticides and pyrethroids; RR, rate ratio.

## Data Availability

Select de-identified data are available via ECHO. Please see data availability statement in manuscript for more information.

## Appendix A. Supplementary data

Supplementary data to this article can be found online at https://doi.org/10.1016/j.envint.2026.110488.
