# Supplementary material for: Prenatal exposure to a mixture of non-persistent endocrine-disrupting chemicals and attention and hyperactivity symptoms in children in the ECHO cohort
Source: Environ Int. Author manuscript; Available in PMC 2026 Oct 22. (PMC13689746; doi:10.1016/j.envint.2026.110488)
Supplement: Supplementary file 1 [file NIHMS2211661-supplement-1.docx]

Supplementary Material

Supplementary Figure 1. Inclusion and exclusion chart

Supplementary Figure 2. Directed Acyclic Graph

Supplementary Figure 3. Spearman Correlation Heatmap

Supplementary Figure 4. Butterfly chart of quantile g-computation model weights for molar sum group components for preschool-aged CBCL

Supplementary Figure 5. Butterfly chart of quantile g-computation model weights for molar sum group components school-aged CBCL

Supplementary Figure 6. Butterfly chart of quantile g-computation model weights for analyte components for preschool-aged CBCL

Supplementary Figure 7. Butterfly chart of quantile g-computation model weights for analyte components for school-aged CBCL

Supplementary Figure 8. Associations between prenatal exposure to a mixture of endocrine-disrupting chemicals and Attention Deficit and Hyperactivity Problem Scores in minimally adjusted models

Supplementary Table 1. Study Analytes and Non-Detection Rates

Supplementary Table 2. Study Sample Characteristics by CBCL form

Supplementary Table 3. Chemical Group Characteristics

Supplementary Table 4. Associations between Endocrine-Disrupting Chemical Mixture Composed of Individual Analytes and Child ADHD Symptoms

Supplementary Table 5. Associations between prenatal exposure to a mixture of endocrine-disrupting chemicals and CBCL Attention Deficit and Hyperactivity Problem T-scores and Parent Reported ADHD Diagnosis

Supplementary Table 6. Associations between Molar Sum Groups and CBCL Raw Scores 85th and 95th Percentiles Thresholds

Supplementary Table 7. Associations between Endocrine-Disrupting Chemical Mixture Composed of Individual Analytes and Child ADHD Symptoms Stratified by age at outcome assessment

Supplementary Table 8. Associations between Endocrine-Disrupting Chemical Mixture Composed of Individual Analytes and Child ADHD Symptoms Stratified by trimester

Supplementary Table 9. Associations between Endocrine-Disrupting Chemical Mixture Composed of Individual Analytes and Child ADHD Symptoms Stratified by Sex

Supplementary Table 10. Associations between Endocrine-Disrupting Chemical Mixture Composed of Individual Analytes and Child ADHD Symptoms Stratified by Child Opportunity Index

Supplementary Table 11. Associations between Endocrine-Disrupting Chemical Mixture Composed of Individual Analytes and Child ADHD Symptoms Excluding Smaller Cohort Sites

Supplementary Table 12. Associations between Endocrine-Disrupting Chemical Mixture Composed of Individual Analytes and Child ADHD Symptoms in Complete Case Analyses

Supplementary Figure 1. Inclusion and exclusion flow chart

Supplementary Figure 2. Directed Acyclic Graph

EDC, endocrine disrupting chemical; BMI, body mass index; ADHD, attention deficit and hyperactivity disorder symptoms

Supplementary Figure 3. Spearman Correlation Heatmap

OPEs, organophosphate esters; OP, organophosphate pesticides and pyrethroids; HMW, high-molecular-weight; LMW, low-molecular-weight; PAH, polycyclic aromatic hydrocarbons; BPS, bisphenol S; Corr, correlation coefficient

Supplementary Figure 4. Butterfly chart of quantile g-computation model weights for molar sum group components for preschool-aged CBCL

Bars represent qgcomp partial weights, reflecting each exposure's relative contribution to the overall mixture effect within the count (symptom count among those with any symptoms) or zero (odds of zero symptoms) component of the hurdle model. Positive (red) and negative (blue) weights sum to 1 and -1, respectively, within each component. OPEs, organophosphate esters; HMW, high-molecular-weight; LMW, low-molecular-weight; PAH, polycyclic aromatic hydrocarbons; BPS, bisphenol S

Supplementary Figure 5. Butterfly chart of quantile g-computation model weights for molar sum group components school-aged CBCL

Bars represent qgcomp partial weights, reflecting each exposure's relative contribution to the overall mixture effect within the count (symptom count among those with any symptoms) or zero (odds of zero symptoms) component of the hurdle model. Positive (red) and negative (blue) weights sum to 1 and -1, respectively, within each component. OPEs, organophosphate esters; HMW, high-molecular-weight; LMW, low-molecular-weight; PAH, polycyclic aromatic hydrocarbons; BPS, bisphenol S

Supplementary Figure 6. Butterfly chart of quantile g-computation model weights for analyte components for preschool-aged CBCL

Bars represent qgcomp partial weights for individual biomarkers, reflecting each exposure's relative contribution to the overall mixture effect within the count (symptom count among those with any symptoms) or zero (odds of zero symptoms) component of the hurdle model. Positive (red) and negative (blue) weights sum to 1 and -1, respectively, within each component. Full biomarker names and abbreviations are provided in Supplementary Table 1.

Supplementary Figure 7. Butterfly chart of quantile g-computation model weights for analyte components for school-aged CBCL

Bars represent qgcomp partial weights for individual biomarkers, reflecting each exposure's relative contribution to the overall mixture effect within the count (symptom count among those with any symptoms) or zero (odds of zero symptoms) component of the hurdle model. Positive (red) and negative (blue) weights sum to 1 and -1, respectively, within each component. Full biomarker names and abbreviations are provided in Supplementary Table 1.

Supplementary Figure 8. Associations between prenatal exposure to a mixture of endocrine-disrupting chemicals and Attention Deficit and Hyperactivity Problem Scores in minimally adjusted models

RR = rate ratio for ADHD raw score among children with a non-zero score per one-quartile joint increase in all exposures in the mixture derived from negative binomial hurdle mixture models.

OR = odds ratio for the likelihood of exhibiting ADHD symptoms (score not = 0) per one-quartile joint increase in all exposures in the mixture derived from negative binomial hurdle mixture models.

Minimally adjusted models included maternal age, body mass index, parity, child sex and age at CBCL assessment, family history of psychiatric illness, and cohort site. Fully adjusted models included maternal age, body mass index, education, parity, pregnancy smoking and alcohol use, race, ethnicity, and child sex and age at CBCL assessment, family history of psychiatric illness, and cohort site. Analyses were conducted separately among preschool-age (n=1,942) and school-age (n=2,020) children

Mixture components included 6 summed groups of: OPEs, organophosphate esters; Pesticides organophosphate pesticides and pyrethroids; PAH, polycyclic aromatic hydrocarbons; HMW Phthalates, high-molecular-weight; LMW Phthalates, low-molecular weight; and Bisphenol S. Chemicals were adjusted for specific gravity and log transformed.

ADHD, attention deficit and hyperactivity disorder; CBCL, Child Behavior Checklist; CI, confidence interval; RR, rate ratio; OR, odds ratio

| Supplementary Table 1. Study Analytes and Non-Detection Rates | | | | | |
| --- | --- | --- | --- | --- | --- |
| Assay Class Name | *Analyte Name* | *Analyte Abbreviation* | *% LLOD* | *Geometric mean (ngml)* | |
| Alkyl Phosphate Pesticides (Organophosphorus  Insecticides) and Pyrethroids | 3,5,6-trichloro-2-pyridinol | TCP | 0.5% | | 0.75 |
|  | 3-phenoxybenzoic acid | PBA | 3.1% | | 0.31 |
|  | Trans-3-(2,2-Dichlorovinyl)-2,2-dimethylcyclopropane carboxylic acid | Trans-DCCA | 21.2% | | 0.27 |
|  | cis-3-(2,2-Dichlorovinyl)-2,2-dimethylcyclopropane carboxylic acid | cis-DCCA | 69.6% | | - |
|  | 4-Fluoro-3-phenoxybenzoic acid | FPBA | 86.6% | | - |
| Organophosphorus Flame Retardants | Bis(1,3-dichloro-2-propyl) phosphate | BDCPP | 13.0% | | 0.49 |
|  | Diphenyl phosphate | DPhP | 17.9% | | 1.0 |
|  | Triethyl phosphate | TEP | 67.3% | | - |
|  | Triphenyl phosphate | TPhP | 78.9% | | - |
|  | Composite of Dibutyl phosphate and Di-isobutyl phosphate | DBuP_DiBP | 83.9% | | - |
|  | Tris(2-butoxyethyl) phosphate | TBOEP | 87.8% | | - |
|  | Composite of Tri-n-butyl phosphate and Tri-isobutyl phosphate | TnBP_TiBP | 91.6% | | - |
|  | Di-o-cresylphosphate | DoCP | 95.2% | | - |
|  | Tris(2-chloroethyl) phosphate | TCEtP | 98.1% | | - |
|  | Tripropyl phosphate | TPP | 100.0% | | - |
| Bisphenols | Bisphenol S | BPS | 13.7% | | 0.30 |
|  | Bisphenol F | BPF | 38.5% | | - |
|  | Bisphenol A | BPA | 60.0% | | - |
|  | Bisphenol A diglycidyl ether | BADGE | 87.4% | | - |
|  | Bisphenol AP | BPAP | 89.2% | | - |
|  | Bisphenol P | BPP | 89.8% | | - |
|  | Bisphenol A (2,3-dihydroxypropyl) glycidyl ether | BADGEH2O | 91.1% | | - |
|  | Bisphenol B | BPB | 92.8% | | - |
|  | Bisphenol Z | BPZ | 94.3% | | - |
|  | Bisphenol A bis(2,3-dihydroxypropyl) glycidyl ether | BADGE2H2O | 95.2% | | - |
|  | Bisphenol AF | BPAF | 99.0% | | - |
|  | 3,3',5,5'-Tetrabromobisphenol A | TBBPA3355 | 99.2% | | - |
|  | 2,2',6,6'-Tetrachlorobisphenol A | TECBPA2266 | 100.0% | | - |
|  | 3,3',5-Tetrachlorobisphenol A | TCBPA335 | 100.0% | | - |
| Phthalate and Phthalate Alternatives | Mono (2-ethyl-5-hydroxyhexyl) phthalate | MEHHP | 0.2% | | 7.45 |
|  | Mono (2-ethyl-5-oxohexyl) phthalate | MEOHP | 0.2% | | 3.92 |
|  | Monoethyl phthalate | MEP | 0.2% | | 37.07 |
|  | Composite of mono-isobutyl phthalate and mono-n-butyl phthalate | MnBP MiBP | 0.3% | | 18.82 |
|  | Mono-(2-ethyl-5-carboxypentyl) phthalate | MECPP | 0.9% | | 6.98 |
|  | Mono-2-(propyl-6-carboxy-hexyl)-phthalate | MPCHP | 1.7% | | 0.56 |
|  | Mono-carboxy isooctyl phthalate | MCIOP | 2.0% | | 5.28 |
|  | Mono-carboxy isononyl phthalate | MCINP | 2.5% | | 0.83 |
|  | Mono-benzyl phthalate | MBZP | 3.6% | | 5.45 |
|  | Mono (3-carboxypropyl) phthalate | MCPP | 11.9% | | 0.73 |
|  | Mono(7-carboxyheptyl) phthalate | MCHPP | 17.3% | | 1.51 |
|  | Mono-2-(propyl-6-oxoheptyl)-phthalate | MPOHP | 17.8% | | 0.26 |
|  | Mono-2-(carboxymethyl) hexyl phthalate | MCMHP | 18.1% | | 1.19 |
|  | Monohydroxy-iso-nonyl phthalate | MHINP | 26.1% | | 0.45 |
|  | Mono ethyl hexyl phthalate | MEHP | 29.4% | | 0.79 |
|  | Cyclohexane-1,2-dicarboxylic acid mono hydroxyisononyl ester | MHNCH | 35.1% | | - |
|  | mono-2-heptyl phthalate | MHPP | 36.1% | | - |
|  | mono-methyl phthalate | MMP | 37.6% | | - |
|  | Cyclohexane-1,2-dicarboxylic acid-mono(oxo-isononyl) ester | MONCH | 40.9% | | - |
|  | mono-hexyl phthalate | MHXP | 43.0% | | - |
|  | Cyclohexane-1,2-dicarboxylic acid mono carboxyisooctyl ester | MCOCH | 52.9% | | - |
|  | Mono-2-(propyl-6-hydroxy-heptyl)-phthalate | MPHHP | 69.6% | | - |
|  | Mono-isopropyl phthalate | MIPP | 73.1% | | - |
|  | Monohydroxy-iso-decyl phthalate | MHiDP | 80.3% | | - |
|  | phthalic acid | PA | 86.9% | | - |
|  | mono-isodecyl phthalate | MIDP | 92.6% | | - |
|  | Mono-propyl phthalate | MPrP | 92.8% | | - |
|  | Mono-n-octyl phthalate | MOP | 95.0% | | - |
|  | Mono-ethyl terephthalate | METP | 96.0% | | - |
|  | mono-pentyl phthalate | MPEP | 97.0% | | - |
|  | Mono-tert-butyl terephthalate | MTBTP | 98.4% | | - |
|  | Mono-benzyl terephthalate | MBzTP | 99.8% | | - |
| Polycyclic Aromatic Hydrocarbons | 2-hydroxynaphthalene | NAP2 | 1.2% | | 0.93 |
|  | 1-hydroxynaphthalene | NAP1 | 21.9% | | 3.58 |
|  | Composite of 1-hydroxphenanthrene and 9-hydroxyphenanthrene | PHEN1 PHEN9 | 29.8% | | 0.53 |
|  | 1-hydroxypyrene | PYR1 | 32.3% | | - |
|  | 2-hydroxyphenanthrene + 3-hydroxyphenanthrene | PHEN2PHEN3 | 48.6% | | - |
|  | Composite of 2-hydroxyfluorene, 3-hydroxyfluorene, and 9-hydroxyfluorene | FLUOs | 54.7% | | - |
|  | 4-hydroxyphenanthrene | PHEN4 | 82.5% | | - |
| Note: Phthalates were grouped further as either low molecular weight (MEP, MnBP, MiBP) or high molecular weight (MEHHP, MEOHP, MECPP, MPCHP, MCIOP, MCINP, MBZP, MCPP, MCHPP, MPOHP, MCMHP, MHINP, MEHP)*. Polycyclic aromatic hydrocarbons were also grouped (NAP1, NAP2, PHEN1PHEN9). Analytes that exceeded 30% of values being LLOD were excluded from primary analysis.  *Di(2-ethylhexyl) phthalate or DEHP metabolites (MEHHP, MEOHP, MECPP, MEHP) were summed prior to inclusion in the high molecular weight phthalate group. The geometric mean of DEHP was 0.07  %LLOD, Percent of values less than the limit of detection; ngml, nanograms per milliliter | | | | | |

| **Supplementary Table 2.** Study Sample Characteristics by CBCL form | | | |
| --- | --- | --- | --- |
| *Maternal Characteristics* | | | |
|  |  | Preschool aged  n=1942 | School-aged  N=2020 |
| Maternal Age, year | Mean (SD) | 30.8 (5.5) | 29.3 (5.5) |
| Parity, n (%) | Parous | 1049 (58.1) | 1206 (62.3) |
| Ethnicity, n (%) | Hispanic | 836 (21.1) |  |
| Race, n (%) | American Indian | <10 (<2) | <5 (<1) |
|  | Asian | 131 (7.8) | 70 (4.2) |
|  | Black | 295 (17.6) | 520 (31.2) |
|  | Multiple Race | 75(4.5) | 91 (5.5) |
|  | Pacific Islander | <20 (<2) | <5 (<1) |
|  | White | 1147 (68.3) | 977 (58.6) |
| Education, n (%) | High school or less | 426 (26.5) | 586 (30.8) |
|  | Some college or above | 1180 (73.5) | 1317 (69.2) |
| Income, n (%) | <$50,000 | 606 (42.6) | 789 (52.8) |
|  | $50,000 + | 818(57.4) | 706 (47.2) |
| Smoking during pregnancy, n (%) | No | 1611 (94.3) | 1554 (91.6) |
| Any alcohol use during pregnancy, n (%) | No | 1226 (86.1) | 1309 (78.9) |
| Body mass index, n (%) | Underweight | 45 (2.5) | 59 (3.1) |
|  | Average | 786 (43.4) | 838 (44.5) |
|  | Overweight | 479 (26.4) | 460 (24.4) |
|  | Obese | 502 (27.7) | 528 (28.0) |
| *Child characteristics* | | |  |
| Child sex, female |  | 961 (49.5) | 1001 (49.6) |
| Age at CBCL survey, years | Mean (SD) | 3.6 (1.1) | 8.7 (2.1) |
| Family History Psychiatric illness | Yes | 443 (48.4) | 941 (57.7) |
| *Outcome characteristics* | | |  |
| CBCL Raw Score | Median [IQR] | 3.0 [1.0, 5.0] | 2.0 [1.0, 5.0] |
| CBCL Raw Score P85, n (%) | ≥ 85^th^ percentile threshold | 361 (18.5) | 436 (21.5) |
| CBCL Raw Score P95, n (%) | ≥ 95^th^ percentile threshold | 137 (7.0) | 102 (5.0) |
| Notes: For selected dichotomous variables (parity, ethnicity, any alcohol use during pregnancy, smoking during pregnancy, family history of psychiatric illness, child sex, raw score percentiles, and ADHD diagnosis), only one category and missingness are presented for brevity. Family history of psychiatric illness includes any first-degree relative diagnosed with depression, anxiety, autism, or ADHD. CBCL indicates Child Behavior Checklist. ADHD diagnosis refers to a prior physician diagnosis of ADHD as reported by the caregiver.  Missing data (n (%)): education, n=453 (11.4%); race, n=622 (15.7%); ethnicity, n=178 (4.4%); parity, n=221 (5.6%); any alcohol use during pregnancy, n=866 (21.9%); smoking during pregnancy, n=557 (14.1%); income, n= 1043 (26.3%); Body mass index, n=265 (6.7%); family history of psychiatric illness, n= 1443 (36.4%); parent-reported ADHD diagnosis, n= 2437 (61.5%). | | | |

| Supplementary Table 3. Chemical Group Characteristics | | | | |
| --- | --- | --- | --- | --- |
|  | Geometric Mean | IQR Value | 25^th^ Percentile | 75^th^ Percentile |
| Bisphenol S | 1.4 | 2.5 | 0.6 | 3.14 |
| HMW Phthalates | 217.7 | 340.7 | 100.3 | 441.0 |
| LMW Phthalates | 375.0 | 565.2 | 166.6 | 731.8 |
| PAH | 52.1 | 68.0 | 27.5 | 95.6 |
| OPE | 7.7 | 9.6 | 3.9 | 13.6 |
| OP | 9.5 | 9.8 | 5.3 | 15.1 |
| Note: Values are in nmol/L and pre log transformation.  OPEs, organophosphate esters; OP, organophosphate pesticides and pyrethroids; HMW, high-molecular-weight; LMW, low-molecular-weight; PAH, polycyclic aromatic hydrocarbons; IQR, interquartile range | | | | |

| Supplementary Table 4. Associations between Endocrine-Disrupting Chemical Mixture Composed of Individual Analytes and Child ADHD Symptoms | |
| --- | --- |
| A. Zero Component (Odds of a Zero Score*) | |
| CBCL Type | OR (95%CI) |
| Preschool | 0.97 (0.76, 1.23) |
| School | 0.95 (0.77, 1.16) |
| B. Count Component (Symptom Severity Among Children with Any Symptoms†) | |
| CBCL Type | RR (95%CI) |
| Preschool | 0.96 (0.89, 1.04) |
| School | 1.00 (0.91, 1.09) |
| *OR = odds ratio for the likelihood of exhibiting no ADHD symptoms (score = 0) per one-quartile joint increase in all exposures in the mixture, derived from negative binomial hurdle mixture models.  †RR = rate ratio for ADHD raw score counts among children with a non-zero score per one-quartile joint increase in all exposures in the mixture, derived from negative binomial hurdle mixture models.  All models were adjusted for maternal age, body mass index, education, parity, smoking and alcohol use during pregnancy, race, ethnicity, child sex and age at CBCL assessment, family history of psychiatric illness, and cohort site.  Mixture components included individual analytes: TCP, PBA, Trans-DCCA, BDCPP, DPhP, BPS, MEP, MnBP, MiBP, MPCHP, MCIOP, MCINP, MBZP, MCPP, MCHPP, MPOHP, MCMHP, MHINP, DEHP, NAP2, NAP1, PHEN1, and PHEN9.  Analyses were conducted separately among preschool-age (n=1,942) and school-age (n=2,020) children. ADHD symptoms were derived from the ADHD subscales of the Child Behavior Checklist (preschool and school-age versions). Chemicals were adjusted for specific gravity and log-transformed.  ADHD, attention deficit and hyperactivity disorder; CBCL, Child Behavior Checklist; CI, confidence interval; CBCL, Child Behavior Checklist; CI, confidence interval | |

| Supplementary Table 5. Associations between prenatal exposure to a mixture of endocrine-disrupting chemicals and CBCL Attention Deficit and Hyperactivity Problem T-scores and Parent Reported ADHD Diagnosis | | | |
| --- | --- | --- | --- |
| Outcome | Sample | Unadjusted OR* (95% CI) | Adjusted OR* (95% CI) |
| CBCL T-score cutoff <65 vs. >65 | Preschool | 1.72 (1.09, 2.73) | 1.31 (0.76, 2.23) |
|  | School | 0.99 (0.75, 1.31) | 0.91 (0.67, 1.24) |
| Parent-report ADHD Diagnosis | Subsample (n= 2,637) | 1.31 (1.00, 1.73) | 1.06 (0.78, 1.45) |
| Outcome | Sample | Unadjusted Estimate† (95% CI) | Adjusted Estimate† (95% CI) |
| Continuous CBCL T-scores | Preschool | 0.46 (0.14, 0.76) | 0.11 (-0.24, 0.46) |
|  | School | -0.18 (-0.66, 0.31) | -0.32 (-0.82, 0.18) |
| * OR = odds ratio for the likelihood of ADHD symptoms per one-quartile joint increase in all exposures in the mixture.  †Estimates represent change in ADHD T-score counts per one-quartile joint increase in all exposures in the mixture.  Mixture components included 6 summed groups: OPEs, organophosphate esters; Pesticides, organophosphate pesticides and pyrethroids; PAH, polycyclic aromatic hydrocarbons; HMW Phthalates, high-molecular-weight phthalates; LMW Phthalates, low-molecular-weight phthalates; and Bisphenol S.  All models adjusted for maternal age, body mass index,, education, partnership, pregnancy smoking and alcohol use, race, ethnicity, and child sex and age at ADHD assessment (for CBCL outcomes), family history of psychiatric illness, and cohort site. T-score models were not controlled for child sex or age.  Analyses were conducted separately among preschool-age (n=1,942) and school-age (n=2,020) children. ADHD symptoms were derived from the ADHD subscales of the Child Behavior Checklist (preschool and school-age versions). Chemicals were adjusted for specific gravity and log-transformed.  ADHD, attention deficit and hyperactivity, CBCL, Child Behavior Checklist; CI, confidence interval. | | | |

| Supplementary Table 6. Associations between Molar Sum Groups and CBCL Raw Scores 85th and 95th Percentiles Thresholds | | | | |
| --- | --- | --- | --- | --- |
|  | Odds of Scores ≥ 85th percentile | | Odds of Scores ≥ 95th percentile | |
|  | *CBCL/1½-5* | *CBCL/6-18* | *CBCL/1½-5* | *CBCL/6-18* |
| HMW Phthalates | 1.08 (0.93, 1.25) | 1.17 (0.93, 1.47) | 1.05 (0.89, 1.23) | 0.98 (0.76, 1.25) |
| LMW Phthalates | 1.01 (0.89, 1.14) | 0.98 (0.80, 1.18) | 0.98 (0.88, 1.10) | 0.97 (0.79, 1.20) |
| Bisphenol S | 1.02 (0.88, 1.18) | 1.01 (0.80, 1.27) | 0.95 (0.86, 1.06) | 0.99 (0.83, 1.18) |
| PAH | 0.92 (0.8, 1.07) | 0.99 (0.79, 1.23) | 0.93 (0.82, 1.06) | 1.00 (0.79, 1.28) |
| OPEs | 1.01 (0.88, 1.16) | 1.18 (0.95, 1.45) | 0.97 (0.85, 1.12) | 1.09 (0.86, 1.39) |
| OPs | 0.97 (0.84, 1.12) | 1.08 (0.85, 0.36) | 0.84 (0.72, 0.98) | 0.83 (0.62, 1.09) |
| OR = odds ratio for the likelihood of ADHD symptom scores ≥ 85^th^ or 95th percentile per one-quartile joint increase in all exposures in the mixture.  Mixture components included 6 summed groups: OPEs, organophosphate esters; Pesticides, organophosphate pesticides and pyrethroids; PAH, polycyclic aromatic hydrocarbons; HMW Phthalates, high-molecular-weight phthalates; LMW Phthalates, low-molecular-weight phthalates; and Bisphenol S.  All models were run separately and adjusted for maternal age, body mass index, education, parity, smoking and alcohol use during pregnancy, race, ethnicity, child sex and age at CBCL assessment, family history of psychiatric illness, and cohort site. Analyses were conducted separately among preschool-age (n=1,942) and school-age (n=2,020) children. ADHD symptoms were derived from the ADHD subscales of the CBCL (preschool and school-age versions). Chemicals were adjusted for specific gravity and log-transformed.  ADHD, attention deficit and hyperactivity disorder; CBCL, Child Behavior Checklist; CI, confidence interval; HMW, high molecular weight; LMW, low molecular weight; OPEs, organophosphate esters; PAH, polycyclic aromatic hydrocarbons; Pesticides, organophosphate pesticides and pyrethroids; RR, rate ratio. | | | | |

| Supplementary Table 7. Associations between Endocrine-Disrupting Chemical Mixture Composed of Individual Analytes and Child ADHD Symptoms Stratified by Age at Outcome Assessment | | |
| --- | --- | --- |
| A. *Preschool-aged CBCL* | | |
|  | Zero Component | Count Component |
| Ages (years) | OR(95%CI) | RR (95%CI) |
| *1½ – 3 (n=840)* | 1.04 (0.74, 1.47) | 0.99 (0.9, 1.08) |
| *4-5 (n=1102)* | 0.89 (0.68, 1.17) | 1.04 (0.94, 1.15) |
| B. *School-aged CBCL* | | |
| *6-9 (n=1599)* | 1.07 (0.86, 1.34) | 0.96 (0.87, 1.05) |
| 10-18 (n=421) | 0.77 (0.51, 1.18) | 0.98 (0.79, 1.22) |
| * OR = odds ratio for the likelihood of exhibiting no ADHD symptoms (score = 0) per one-quartile joint increase in all exposures in the mixture. † RR = rate ratio for ADHD raw score counts among children with a non-zero score per one-quartile joint increase in all exposures in the mixture.  Mixture components included 6 summed groups: OPEs, organophosphate esters; Pesticides, organophosphate pesticides and pyrethroids; PAH, polycyclic aromatic hydrocarbons; HMW Phthalates, high-molecular-weight phthalates; LMW Phthalates, low-molecular-weight phthalates; and Bisphenol S.  Among preschool-aged (n=1942), n=840 were 1½-3 year olds and n=1102 were 4-5 year olds. Among school-aged (n=2020), n=1599 were 6-9 year olds and n=421 year olds. Analyses were conducted separately among preschool-age (n=1,942) and school-age (n=2,020) children.  All models adjusted for maternal age, body mass index, education, smoking, alcohol, race, ethnicity, child age, family history of psychiatric illness, and cohort site. ADHD symptoms were derived from the ADHD subscales of the CBCL (preschool and school-age versions). Chemicals were adjusted for specific gravity and log-transformed.  ADHD, attention deficit and hyperactivity disorder; CBCL, Child Behavior Checklist; CI, confidence interval. | | |

| Supplementary Table 8. Associations between Endocrine-Disrupting Chemical Mixture Composed of Individual Analytes and Child ADHD Symptoms Stratified by Trimester | | |
| --- | --- | --- |
| A. *Preschool-aged CBCL* | | |
|  | Zero Component | Count Component |
| Trimester of Sample Collection | OR (95%CI) | RR (95%CI) |
| 2^nd^ trimester | 0.96 (0.71, 1.3) | 1.06 (0.96, 1.16) |
| 3^rd^ trimester | 0.81 (0.58, 1.12) | 0.92 (0.82, 1.03) |
| B. *School-aged CBCL* | | |
| 2^nd^ trimester | 0.98 (0.76, 1.26) | 0.94 (0.84,1.05) |
| 3^rd^ trimester | 0.95 (0.71, 1.27) | 1.06 (0.91,1.23) |
| * OR = odds ratio for the likelihood of exhibiting no ADHD symptoms (score = 0) per one-quartile joint increase in all exposures in the mixture. † RR = rate ratio for ADHD raw score counts among children with a non-zero score per one-quartile joint increase in all exposures in the mixture.  Mixture components included 6 summed groups: OPEs, organophosphate esters; Pesticides, organophosphate pesticides and pyrethroids; PAH, polycyclic aromatic hydrocarbons; HMW Phthalates, high-molecular-weight phthalates; LMW Phthalates, low-molecular-weight phthalates; and Bisphenol S.  Analyses were conducted separately among preschool-age and school-age children by trimester of sample collection. Among preschool-age children, n=1,032 had 2nd trimester samples, and n=716 had 3rd trimester samples. Among school-age children, n=1,046 had 2nd trimester samples, and n=794 had 3rd trimester samples.  All models adjusted for maternal age, body mass index, education, smoking, alcohol, race, ethnicity, child age, family history of psychiatric illness, and cohort site. ADHD symptoms were derived from the ADHD subscales of the CBCL (preschool and school-age versions). Chemicals were adjusted for specific gravity and log-transformed.  ADHD, attention deficit and hyperactivity disorder; CBCL, Child Behavior Checklist; CI, confidence interval. | | |

| Supplementary Table 9. Associations between Endocrine-Disrupting Chemical Mixture Composed of Individual Analytes and Child ADHD Symptoms Stratified by Sex | | |
| --- | --- | --- |
| A. Zero Component (Odds of a Zero Score*) | | |
|  | Female | Male |
| CBCL Type | OR (95%CI) | |
| Preschool | 0.88 (0.66, 1.17) | 1.03 (0.74, 1.43) |
| School | 1.06 (0.81, 1.39) | 0.88 (0.65, 1.18) |
| B. Count Component (Symptom Severity Among Children with Any Symptoms†) | | |
|  | Female | Male |
| CBCL Type | RR (95%CI) | |
| Preschool | 1.04 (0.94, 1.15) | 1.01 (0.92, 1.10) |
| School | 0.91 (0.80, 1.04) | 1.02 (0.91, 1.14) |
| * OR = odds ratio for the likelihood of exhibiting no ADHD symptoms (score = 0) per one-quartile joint increase in all exposures in the mixture. † RR = rate ratio for ADHD raw score counts among children with a non-zero score per one-quartile joint increase in all exposures in the mixture.  All models adjusted for maternal age, body mass index, education, smoking, alcohol, race, ethnicity, child age, family history of psychiatric illness, and cohort site.  Among females (n=1962), n=961 had preschool and n=1001 had school forms. Among males (n=2000), n=981 had preschool and n=1019 had school forms.  Mixture components included 6 summed groups: OPEs, organophosphate esters; Pesticides, organophosphate pesticides and pyrethroids; PAH, polycyclic aromatic hydrocarbons; HMW Phthalates, high-molecular-weight phthalates; LMW Phthalates, low-molecular-weight phthalates; and Bisphenol S. ADHD symptoms were derived from the ADHD subscales of the CBCL (preschool and school-age versions). Chemicals were adjusted for specific gravity and log-transformed.  ADHD, attention deficit and hyperactivity disorder; CBCL, Child Behavior Checklist; CI, confidence interval. | | |

| Supplementary Table 10. Associations between Endocrine-Disrupting Chemical Mixture Composed of Individual Analytes and Child ADHD Symptoms Stratified by Child Opportunity Index | | | | | |
| --- | --- | --- | --- | --- | --- |
| Zero Component (Odds of a Zero Score*) | | | | | |
|  | Very Low COI | Low COI | Moderate COI | High COI | Very High COI |
| Preschool + School CBCL | 0.99 (0.74, 1.33) | 0.82 (0.50, 1.35) | 1.16 (0.76, 1.77) | 0.84 (0.58, 1.20) | 1.06 (0.79, 1.43) |
| Count Component (RR† ) | | | | | |
| Preschool + School CBCL | 1.06 (0.95, 1.18) | 1.05 (0.90, 1.22) | 1.10 (0.95, 1.28) | 1.13 (0.99, 1.29) | 0.88 (0.79, 0.99) |
| * OR = odds ratio for the likelihood of exhibiting no ADHD symptoms (score = 0) per one-quartile joint increase in all exposures in the mixture. † RR = rate ratio for ADHD raw score counts among children with a non-zero score per one-quartile joint increase in all exposures in the mixture.  Please note due to statistical power constraints we combined these analyses. All models were adjusted for maternal age, body mass index, education, parity, smoking and alcohol use during pregnancy, race, ethnicity, child sex and age at CBCL assessment, family history of psychiatric illness and cohort site. Analyses were conducted in the full sample (preschool and school-age children combined; n=3,962) to preserve statistical power.  Mixture components included 6 summed groups: OPEs, organophosphate esters; Pesticides, organophosphate pesticides and pyrethroids; PAH, polycyclic aromatic hydrocarbons; HMW Phthalates, high-molecular-weight phthalates; LMW Phthalates, low-molecular-weight phthalates; and Bisphenol S. ADHD symptoms were derived from the ADHD subscales of the CBCL (preschool and school-age versions). Chemicals were adjusted for specific gravity and log transformed.  CBCL, Child Behavior Checklist; CI, confidence interval; COI, Child Opportunity Index, RR, rate ratio; OR, odds ratio; | | | | | |

| Supplementary Table 11. Prenatal exposure to a mixture of endocrine disrupting chemicals and child ADHD symptoms excluding Smaller Cohort Sites | |
| --- | --- |
| A. Zero Component (Odds of a Zero Score*) | |
| CBCL Type | OR (95% CI) |
| Preschool | 0.93 (0.74, 1.16) |
| School | 0.95 (0.78, 1.17) |
| B. Count Component (Symptom Severity Among Children with Any Symptoms†) | |
| CBCL Type | RR (95% CI) |
| Preschool | 1.04 (0.97, 1.11) |
| School | 0.96 (0.88, 1.05) |
| * OR = odds ratio for the likelihood of exhibiting no ADHD symptoms (score = 0) per one-quartile joint increase in all exposures in the mixture.  † RR = rate ratio for ADHD raw score counts among children with a non-zero score per one-quartile joint increase in all exposures in the mixture.  A total of n=271 participants were dropped from sites with fewer than n=100 participants. Analyses were then conducted separately among preschool-age (n=1777) and school-age children (n=2020).  Mixture components included 6 summed groups: OPEs, organophosphate esters; Pesticides, organophosphate pesticides and pyrethroids; PAH, polycyclic aromatic hydrocarbons; HMW Phthalates, high-molecular-weight phthalates; LMW Phthalates, low-molecular-weight phthalates; and Bisphenol S.  All models adjusted for maternal age, body mass index, education, smoking, alcohol, race, ethnicity, child age, family history of psychiatric illness, and cohort site. ADHD symptoms were derived from the ADHD subscales of the CBCL (preschool and school-age versions). Chemicals were adjusted for specific gravity and log transformed.  ADHD, attention deficit and hyperactivity disorder; CBCL, Child Behavior Checklist; CI, confidence interval. | |

| Supplementary Table 12. Prenatal exposure to a mixture of endocrine disrupting chemicals and child ADHD symptoms: Complete case analysis | |
| --- | --- |
| A. Zero Component (Odds of a Zero Score*) | |
| CBCL Type | OR (95% CI) |
| Preschool | 1.32 (0.78, 2.25) |
| School | 0.96 (0.71, 1.30) |
| B. Count Component (Symptom Severity Among Children with Any Symptoms†) | |
| CBCL Type | RR (95% CI) |
| Preschool | 1.08 (0.82, 1.28) |
| School | 0.89 (0.79, 1.00) |
| * OR = odds ratio for the likelihood of exhibiting no ADHD symptoms (score = 0) per one-quartile joint increase in all exposures in the mixture.  † RR = rate ratio for ADHD raw score counts among children with a non-zero score per one-quartile joint increase in all exposures in the mixture.  Analyses were conducted among those with complete information for all variables in preschool-age (n=363) and school-age children (n=878).  Mixture components included 6 summed groups: OPEs, organophosphate esters; Pesticides, organophosphate pesticides and pyrethroids; PAH, polycyclic aromatic hydrocarbons; HMW Phthalates, high-molecular-weight phthalates; LMW Phthalates, low-molecular-weight phthalates; and Bisphenol S.  All models adjusted for maternal age, body mass index, education, smoking, alcohol, race, ethnicity, child age, family history of psychiatric illness, and cohort site. ADHD symptoms were derived from the ADHD subscales of the CBCL (preschool and school-age versions). Chemicals were adjusted for specific gravity and log transformed.  ADHD, attention deficit and hyperactivity disorder; CBCL, Child Behavior Checklist; CI, confidence interval. | |
